# Psychometric Evaluation of the German Version of the Perceived Access to Healthcare Questionnaire in a Sample of Individuals with Rare Chronic Diseases

**DOI:** 10.3390/healthcare12060661

**Published:** 2024-03-15

**Authors:** Susanne Wehrli, Andrew A. Dwyer, Markus A. Landolt

**Affiliations:** 1 Department of Psychosomatics and Psychiatry, University Children’s Hospital, University of Zurich, 8032 Zurich, Switzerland; 2 Division of Child and Adolescent Health Psychology, Department of Psychology, University of Zurich, 8032 Zurich, Switzerland; 3 Children’s Research Center, University Children’s Hospital, University of Zurich, 8032 Zurich, Switzerland; 4 University Research Priority Program “ITINERARE–Innovative Therapies in Rare Disease”, University of Zurich, 8032 Zurich, Switzerland; 5 William F. Connell School of Nursing, Boston College, Chestnut Hill, Boston, MA 02467, USA; 6 P50 Massachusetts General Hospital—Harvard Center for Reproductive Medicine Boston, Boston, MA 02114, USA

**Keywords:** healthcare access, health services, rare disease, chronic disease, rare disease, psychometric evaluation

## Abstract

Access to healthcare is multifaceted and poses significant challenges for individuals with chronic and rare diseases (RDs). This study aimed to conduct a psychometric evaluation of the German version of the Perception of Access to Healthcare Questionnaire (PAHQ) among individuals with RDs. We conducted an evaluation of the PAHQ using a sample of 271 adults with an RD diagnosis. The 31-item instrument underwent evaluation including a comparison of three different confirmatory factor models (CFA). Subsequent steps involved item removal, reliability analysis (computation of Cronbach’s alpha), and analysis of criterion-related validity. The six-factor model showed the best fit to the data and was selected for further examination. Subsequently, six items were removed. Fit indices for the final model were acceptable. Cronbach’s alpha ranged from 0.75 to 0.91 for the six subscales, except for the availability subscale which exhibited the lowest value (0.64). In terms of criterion-related validity, different skills relating to the navigation of access dimensions were significantly correlated with corresponding PAHQ subscales, thus confirming validity. The capacity of the PAHQ to guide targeted interventions and facilitate cross-population comparisons positions it as a valuable instrument for advancing healthcare access research and promoting equitable access to care, particularly for individuals with rare and chronic diseases.

## 1. Introduction

Access to healthcare is widely recognized as a fundamental and basic human right. However, individuals with chronic health conditions often face challenges in accessing healthcare services. Individuals with rare diseases (RDs) are confronted with particular obstacles regarding access to healthcare. Notably, RDs are complex conditions that are typically chronic, progressive, and have significant morbidity and mortality [1]. Although individual RDs are uncommon, there are approximately 7000 RDs [2]. Thus, the cumulative number of individuals affected by RDs is substantial. Recent estimates indicate that 3.5 to 5.9% of the global population is diagnosed with an RD (263–446 million individuals) [3,4]. 

Individuals with RDs often face extended diagnostic journeys, encounter numerous obstacles when seeking healthcare, and face difficulties in accessing affordable care and treatment [5]. In addition, healthcare professionals often lack sufficient knowledge about RDs, contributing to feelings of mistrust, frustration, and anger among affected individuals [6]. The physical, psychological, and economic burdens associated with RDs underscore their importance within the realm of public health [7]. Consequently, research is needed to inform healthcare policies and strategies to improve healthcare access for individuals with RDs. 

The construct “healthcare access” is complex and multifaceted with varying definitions and dimensions [8]. Researchers have approached healthcare access from different perspectives, focusing on utilization, resource availability, or user characteristics [9]. Some models emphasize the factors and characteristics of health services, while others highlight healthcare resources. Historically, access has often been linked to utilization as a proxy implying a unifactorial structure of the construct [10]. Other models, such as the one proposed by Penchansky and Thomas [11], define access as the connection between healthcare users and system resources, highlighting the importance of compatibility between users and providers [12]. The framework includes dimensions of accessibility, availability, acceptability, affordability, and adequacy (also known as accommodation), along with an additional dimension of ‘awareness’ [13].

In 2013, Levesque [14] and colleagues proposed a theoretical model based on the prior work of Pechansky and Thomas [11]. The Levesque model consists of five dimensions of access: approachability, acceptability, availability and accommodation, affordability, and appropriateness. It also incorporates corresponding patient abilities, including the ability to perceive (e.g., health literacy), the ability to seek (e.g., patient activation), the ability to reach (e.g., physical mobility), the ability to engage (e.g., autonomy preference) and the ability to pay (e.g., socioeconomic status). For instance, assessing access involves not only the cost of a specific health service but also considers the ability of individuals receiving the service to afford it (socioeconomic status). The Levesque model is a patient-centered framework that considers the individual (demand side). While the model does not explicitly use the term “fit”, it clearly highlights the dynamic relationship between the characteristics of healthcare recipients and providers. Consequently, the frameworks proposed by Levesque et al. [14] and Penchansky and Thomas [11] differ in terms of the number and content of dimensions as well as the inclusion of individual capabilities. Both frameworks have been successfully applied to various chronic diseases and healthcare systems [15,16,17,18].

Quantifying healthcare access is challenging, often leading to the use of proxies like healthcare utilization that do not fully capture the construct’s complexity and multidimensionality [19]. Access questionnaires have traditionally focused on specific dimensions rather than a comprehensive assessment of access [20]. Additionally, none of the existing scales have been validated. Recently, the Perception of Access to Healthcare Questionnaire (PAHQ) [21] was developed based on Penchansky and Thomas’ model [11] and includes an additional ‘awareness’ dimension [13]. The 30-item PAHQ has been validated for content, construct, and face validity in a sample of healthy Iranian adults [21].

To date, the PAHQ has not been evaluated using alternative factor structures, such as the five-factor structure proposed by Levesque and colleagues [14], or a unidimensional structure based on the historical roots of the construct. The PAHQ has yet to be validated in individuals with chronic diseases or RDs. Given the increasing importance of RDs in healthcare systems and the need to improve healthcare access for RD populations, validated instruments are needed to evaluate healthcare access. We aimed to validate the German version of the PAHQ using confirmatory factor analysis (CFA) to compare the unidimensional-factor structure with both the six-factor structure based on the Penchansky and Thomas [11] model and the five-factor structure proposed by Levesque et al. [14]. Subsequently, criterion-related validity was assessed by comparing the subscales to the abilities outlined by Levesque et al. [14] to determine consistency with the proposed theoretical access dimensions. Last, we evaluated construct validity, criterion-related validity, and reliability through an analysis of internal consistency using Cronbach’s alpha of the selected factor model within an RD cohort. 

## 2. Materials and Methods

### 2.1. Study Design and Data Collection

We conducted an online cross-sectional survey to collect anonymized data from geographically dispersed, German-, French-, English-, and Italian-speaking Swiss patients with an RD. Participants were recruited through Swiss RD patient organizations and through physicians at the University Children’s Hospitals in Zurich, Berne, and Lausanne. Patients were enrolled in the study using a variety of outreach methods, including newsletters, social media posts, and emails sent directly from their doctors. In addition, patient organizations emailed information about the study to their members. Participants received no compensation for their participation. Informed consent was obtained from all participants at the start of the online survey. As the data collection was fully anonymized, no approval was required from the Ethics Committee of the Canton of Zurich, Switzerland. This exemption was formally recognized by the ethics committee through a clarification of responsibilities.

**Figure 1:**
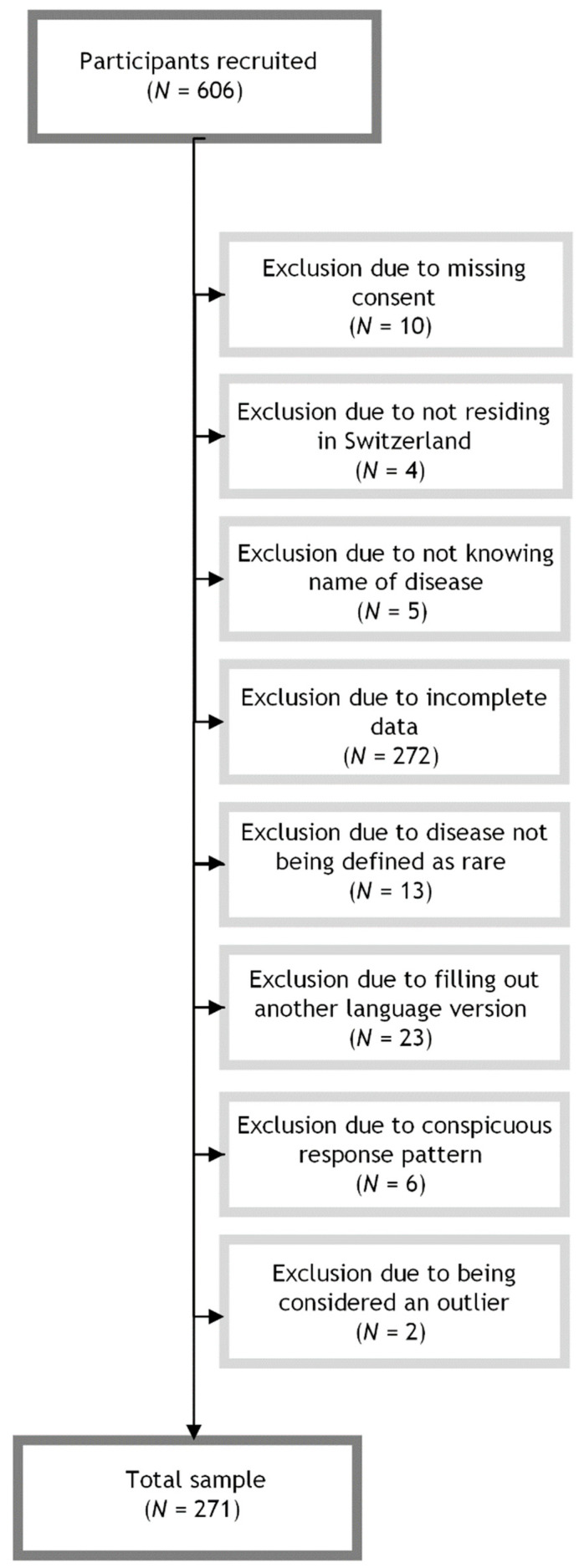
Flowchart of sample inclusion and exclusion.

To be included in the study, participants needed to be at least 18 years old; residing in Switzerland; have sufficient knowledge of German, French, Italian, or English; have a medically confirmed RD diagnosis; and be able to name their respective RD. We defined RD as a disorder affecting fewer than 1 in 2000 people in the European population as delineated in the European Parliament and Council Regulation on Orphan Medicinal Products [22]. A total of 32 participants were excluded from the analysis for disease not meeting RD criteria (*N* = 13), not providing consent (*N* = 10), not knowing the name of their disease (*N* = 5), and not residing in Switzerland (*N* = 4). To ensure criterion-related validity, one skill for each dimension of access was selected for analysis based on the Levesque framework [14]. We excluded participants who did not complete the scales measuring healthcare access skills and those with incomplete PAHQ data (*N* = 272). As the present study specifically focused on the German version of the PAHQ, participants who filled out the English, French, or Italian versions of the study were excluded (*N* = 23). A uniform response pattern was considered an indicator of low-quality survey data and may affect the reliability and validity of the results [23]. Accordingly, surveys that showed signs of careless responses (i.e., answering all PAHQ items identically) were excluded (*N* = 6). Individuals were excluded from the analysis if they were identified as outliers (i.e., falling outside the whiskers of the boxplot). Specifically, data points that fell below the lower whisker (values below Q1 − 1.5 × IQR) or above the upper whisker (values above Q3 + 1.5 × IQR) were considered outliers [24]. Subjects were similarly considered outliers if responses were identified as outliers on at least 25% of PAHQ items. Outliers were removed from the dataset to minimize the potential impact of extreme values on the analysis (*N* = 2). The dataset used for analysis included a total of 271 participants. A flowchart illustrating the exclusion process is provided in Figure 1.

### 2.2. Measures 

#### 2.2.1. Diagnostic Information

Participants self-reported their RD diagnosis. We used the 10th version of the International Statistical Classification of Diseases and Related Health Problems (ICD-10) to group participants into diagnostic categories [25]. Briefly, the ICD-10 is a standardized and widely used system with internal validity and has been successfully used in previous RD studies [26]. When a specific RD diagnosis was not listed in ICD-10 (*N* = 7) the ICD-11 classification system was employed [27].

#### 2.2.2. Access to Healthcare

We used the Perception of Access to Healthcare Questionnaire (PAHQ) to measure healthcare access [21]. The 30-item instrument uses a five-point Likert-type response scale (i.e., 0 “not at all” to 5 “almost always”) for six subscales: (i) availability (e.g., “the facilities of the health center meet the health needs of the clients”), (ii) accessibility (e.g., “ the time required to reach the health center is appropriate”), (iii) affordability (e.g., “cost is a serious barrier to using healthcare”), (iv) adequacy (e.g., “the working hours of the public health center are suitable for receiving services from these centers”), (v) acceptability (e.g., “the quality of services provided in the health center is acceptable”), and (vi) awareness (e.g., “health workers try to make sure I fully understand the health information provided”). To translate and culturally adapt the PAHQ into German, we employed a back-translation method following good best practice principles for the translation and cultural adaptation process for patient-reported outcome measures [28] (see Table S1). One additional item was included (“I have a health professional where all my health information converges”) to address the criticism that the existing frameworks overlook the interconnectedness of multiple providers/specialties involved in managing chronic conditions and the importance of information sharing and care coordination [29].

#### 2.2.3. Ability to Perceive—Health Literacy

Broadly, health literacy refers to the ability to obtain, read, understand, and use healthcare information to make health decisions and follow treatment instructions. Thus, health literacy comprises an individual’s ability to be aware of health services that can meet their needs, knowledge of how to access them, and understanding how services can impact their health [14]. We assessed health literacy using the 12-item European Health Literacy Survey Questionnaire (HLS-Q12) [30] that has been previously used in German-speaking countries, including Switzerland [31]. German translations of the questionnaire were provided by the Federal Office of Public Health [32]. Participants respond to items using a 4-point Likert-type scale (i.e., 1 “very difficult” to 4 “very easy”). Higher scores (sum of 12 items) indicated higher levels of health literacy (α = 0.77).

#### 2.2.4. Ability to Seek—Patient Activation

The ability to seek healthcare refers to an individual’s capacity to autonomously decide to pursue care and their intention to do so [14]. We used the Patient Activation Measure 13 (PAM13) as a proxy for measuring the ability to seek healthcare [33]. The German PAM13 has been previously validated [34,35]. The 13-item instrument uses a 4-point Likert-type scale (i.e., 1 “strongly disagree” to 4 “agree strongly”). Higher scores (sum of 13 items) indicate higher levels of patient activation suggesting a greater likelihood of being actively involved in their health/healthcare decision-making (α = 0.80).

#### 2.2.5. Ability to Reach—Mobility/Physical Functioning

The Short Form Health Survey (SF-12) is a well-validated 12-item questionnaire assessing eight dimensions of health and function (physical functioning, physical role limitation, physical pain, general health, vitality, social functioning, emotional role limitation, and mental health). The results are reported in two summary scores for central factors (mental and physical functioning) [36,37,38]. Summary scores (mental and physical) range from 0 to 100, with higher scores indicating a greater health-related quality of life [36]. The SF-12 has satisfactory reliability and validity [37]. For this study, we only used the physical summary score to capture mobility and thus the ability to reach healthcare (α = 0.86).

#### 2.2.6. Ability to Engage—Autonomy Preference

The ability to engage with healthcare refers to the effectiveness and usefulness of health services when utilized by an individual [14]. In this study, we used the decision-making subscale of the Autonomy Preference Index (API) as a proxy measure for the ability to engage [39]. The German version of the questionnaire was previously validated [40]. The subscale comprises four items. Participants respond using a 5-point Likert-type scale (0 = “strongly disagree”; 4 = “strongly agree”). Relevant subscale item scores are summed to calculate a total score with higher total scores indicating a greater preference for autonomy and active involvement in decision-making regarding one’s health (α = 0.82).

#### 2.2.7. Ability to Pay—Socioeconomic Status

The ability to pay refers to individuals’ capacity to financially access the necessary health services [14]. In this study, education was used as a proxy for socioeconomic status. Participants reported their highest level of education, ranging from non-completion of minimum schooling or special needs education (lowest level) to university education (highest level).

### 2.3. Statistical Analysis

The statistical analysis was conducted using R Statistical Software (version 4.1.2) [41]. Power analyses confirmed that a sufficient number of participants were included (Table S2).

Analyses involved three steps. First, confirmatory factor analysis (CFA) was performed to examine all three possible factor structures using structural equation modeling (SEM) with the lavaan package [42]. The evaluation aimed to assess the construct validity of three models, i.e., the unidimensional-factor model, the five-factor model proposed by Levesque [14], and the six-factor model proposed by Penchansky and Thomas [11]. A comparison was made using the Chi-squared difference test. The final model was used to examine factor loadings. Factor loadings greater than 0.45 (considered ‘fair’) were retained [43]. Loadings below 0.45 have been argued to complicate the interpretation of the latent factor structure as they explain less than 20% of the variance [43]. Due to the strong theoretical underpinnings of the PAHQ, item removal was evaluated in the context of CFA rather than exploratory factor analysis. Only the selected model was retained for further analysis after item removal; the models were no longer nested and, consequently, were not derived from the same data rendering them incomparable. Model fit for the final model was assessed using multiple indices, including comparative fit index (CFI), relative non-centrality index (RNI), root mean square error of approximation (RMSEA), and standard root mean square residual (SRMR) [44]. A CFI ≥0.95 was considered good and a score ≥0.9 was deemed acceptable. An RNI ≥0.90 was defined as good, and values ≥0.08 were considered acceptable. For RMSEA, a value <0.50 was considered good and a value <0.8 was considered acceptable [45]. An SRMR <0.50 was considered good while a value <1.00 was considered acceptable.

Second, we calculated several indicators of reliability, including Cronbach’s alpha (α). Values ≥0.9 were considered excellent, ≥0.80 as good, and ≥0.70 as acceptable [46]. Reliability calculations were conducted using the R package psych [47]. We also calculated ceiling and floor effects for the revised subscales.

Last, non-parametric correlations were examined between the raw scores of the final questionnaire and the scales used to measure the abilities of the corresponding subscales derived by the Levesque et al. [14] model. This step aimed to provide statistical evidence that the PAHQ accurately assesses the intended dimensions of access and thus criterion-related validity. Given that the dimensions of the Levesque et al. [14] model do not directly align with those of the Penchansky and Thomas model [11], the abilities were categorized accordingly. Specifically, the ‘awareness’ subscale was correlated with the API (ability to engage) and the HLSQ-12 (ability to perceive). This choice was made because these subscales encompass items from both the ‘approachability’ and ‘appropriateness’ scales. Furthermore, the ‘availability’, ‘adequacy’, and ‘accessibility’ subscales correlated with the physical component summary score of the SF-12 (ability to reach). These dimensions of the Penchansky and Thomas model [11] correspond to the ‘availability’ and ‘accommodation’ dimensions of the Levesque model. Additionally, the ‘acceptability’ subscale was correlated with both the PAM13 (ability to seek) and the API (ability to engage). This decision was made because the ‘acceptability’ dimension encompasses aspects of both the ‘acceptability’ and ‘appropriateness’ dimensions of the Levesque model.

## 3. Results

### 3.1. Sample Characteristics

Participant characteristics of the total sample are depicted in Table 1. The average age of participants was approximately 48 years. The majority of participants were female and approximately half of the sample had a least a high school education. Similarly, about half of the participants were married. Regarding health-related variables, congenital malformations and endocrine/metabolic diseases were the most frequently reported RD categories. Most individuals reported having a stable disease course.

### 3.2. Confirmatory Factor Analysis (Global Goodness of Fit)

To examine construct validity, we used confirmatory factor analysis (CFA) for the unidimensional-factor model. We also employed CFA for both theoretical models, utilizing the five-factor solution proposed by Levesque et al. [14] and the six-factor solution proposed by Penchansky and Thomas [11]. Between five and six item loadings fell below the a priori cut-off (0.45) (for detailed factor loadings, refer to Table S3). The items were retained for a comparison of the two models. The Chi-square test revealed that the six-factor model demonstrated a superior fit compared to the unidimensional-factor model and the five-factor model (Table 2). Consequently, the six-factor model was selected for further analyses.

### 3.3. Confirmatory Factor Analysis (Local Goodness of Fit) 

Items 1, 13, 14, 19, 25, and 31 from the six-factor model were excluded from the analysis due to their loadings being below the 0.45 threshold (see Table S4 for detailed factor loadings following item removal). After the removal of these six items, loadings for items 17 and 18 were set at 1.0 since the factor in question contained only two items, ensuring the preservation of measurement equivalence. Considering model fit, the CFI (0.869) approached 0.90, indicating an acceptable fit. Similarly, the RMSEA was 0.073 (below the 0.80 threshold), signifying an acceptable fit. The SRMR was 0.084 (below the 1.00 threshold), further indicating an acceptable fit. In addition, the RNI reached 0.840 (above the 0.80 threshold), confirming an acceptable fit.

### 3.4. Internal Consistency

To assess reliability, Cronbach’s alphas were computed for each subscale (Table 3). All subscales but for the ‘availability’ subscale exhibited Cronbach’s alphas within the ‘acceptable’ to ‘excellent’ range [48]. Compared to the previous version of the model (before item removal), the alpha values improved for the ‘accessibility’, ‘acceptability’, ‘affordability’, and ‘awareness’ subscales. This analysis confirms that the items removed are precisely those whose exclusion has a positive effect on Cronbach’s alpha values, thereby improving the overall internal consistency of the scale.

The ceiling and floor effects are detailed in Table 4. Notably, the acceptability subscale showed the strongest floor effect, whereas the highest ceiling effects were observed on the accessibility and affordability subscales.

### 3.5. Criterion-Related Validity 

Pearson correlation coefficients (Table 5) for the PAHQ were significant with all of the corresponding abilities of the Levesque model, indicating a consistent direction of effects.

## 4. Discussion

Herein, we examined the psychometric properties of the German version of the PAHQ, an instrument developed to assess access to healthcare in healthy individuals and to assess its suitability for people with RDs. We conducted CFA to compare three different possible factor solutions, examined the reliability of the German PAHQ, and examined criterion-related validity in a sample of Swiss individuals with RDs. Our analysis indicated that a six-factor model provided the best fit to the data set, necessitating the removal of six items to improve the model fit. The fit indices demonstrated decent fit, and Cronbach’s alpha values were generally adequate across the subscales, with the exception of the availability subscale. Significant correlations were also observed, supporting the criterion-related validity of the instrument. In conclusion, the PAHQ is validated as a reliable and applicable tool for use in populations with chronic diseases.

Examining the structure of the PAHQ, confirmatory factor analysis revealed that the six-factor model, originally proposed by Penchansky and Thomas [11], provides a better fit compared to both the five-factor model posited by Levesque et al. [14] and the unidimensional-factor model. The existing literature demonstrates the complexity of operationalizing access to healthcare. Some definitions emphasize health insurance coverage while others focus on healthcare utilization [8]. The lack of consensus poses a major challenge in measuring healthcare access, particularly in populations with limited access to healthcare and those at risk of having poor access. Previous studies in the field of RDs have not explicitly investigated healthcare access. Rather, prior work has focused on related constructs such as healthcare navigation or has failed to utilize psychometrically valid, theory-derived instruments [49,50,51]. For example, Bambusch and colleagues [49] employed a qualitative approach to examine patient experiences navigating the healthcare system. Molster et al. [50] qualitatively explored patient experiences with healthcare (e.g., diagnostic delays) rather than specifically examining access. Similarly, Navarrette-Opazo et al. [51] centered their investigation on healthcare utilization. It is worth noting that healthcare utilization is frequently employed as an alternative measure of access despite the distinct differences between these two constructs [19]. In summary, past studies have focused on specific, singular dimensions of healthcare access and have failed to capture a comprehensive construct of healthcare access. In the present study, CFA results underscore the value of the six-factor model as opposed to previous unidimensional approaches that depend on availability and utilization as potential clarifiers. Consequently, our findings shed light on the complex construct of healthcare access. Moreover, results suggest that a more comprehensive, multiple-factor model (beyond the one- or five-dimensional versions) is needed to effectively represent healthcare access. Additionally, correlations with corresponding abilities, proposed by Levesque et al. [14], were satisfactory. Therefore, we conclude that the PAHQ is a valid and reliable measure to assess healthcare access in individuals with chronic (and rare) diseases. 

When examining the performance of the ‘affordability’ subscale, item 19 (“cost is a serious barrier to healthcare utilization”) is of particular interest. The internal consistency of the subscale, indicated by Cronbach’s alpha values, improved considerably when item 19 was excluded from the analysis. This observation raises questions regarding item 19’s (cost) contribution to the overall scale and warrants further investigation. The decision to exclude item 19 should not be taken lightly, as it may affect the overall subscale construct and interpretation of the results. One possible explanation for the improved alpha scores after exclusion is the divergent nature of item 19 compared to the other two items in the affordability subscale. Item 19 may be capturing a separate dimension related to financial barriers to healthcare use. Further, it is important to note that items 17 and 18, both of which focus on the steps individuals take when seeking healthcare, indirectly address affordability. Item 17 indicates that individuals typically first see a general practitioner (i.e., primary care provider), possibly due to cost. In contrast, item 18 indicates that individuals seek specialized medical services under the guidance of a general practitioner, reinforcing the notion of cost-driven decision-making. The consideration that item 19 may be measuring a different aspect or dimension related to affordability is relevant. It underscores the need for future research to explore the multifaceted nature of healthcare affordability and the possible presence of sub-dimensions within this broader construct. Moreover, the specific context of the healthcare system under study may play a role in Item 19 performance, and its relevance may vary in settings with different healthcare systems and financial structures. In summary, the removal of item 19 improved the internal consistency of the ‘affordability’ subscale yet raises questions regarding the complexity of the construct.

The removal of “the medical care I need is provided at healthcare facilities” (item 1), “my request for same-sex healthcare staff is being considered” (item 13), “I accept screenings such as cervical and colorectal cancer at healthcare facilities” (item 14), “healthcare facilities provide access to various assistive devices such as wheelchairs, walkers and the like” (item 25), and “I have a medical professional where all my health information comes together” (item 31) could be attributed to the high-quality care provided in Switzerland. Indeed, Swiss healthcare receives high public satisfaction ratings suggesting high-quality care [32,52]. Such observations are often attributed to Switzerland’s consumer-driven healthcare system that combines high-quality care and health equity [53]. Accordingly, the exclusion of certain items (i.e., 1, 13, 14, 25, and 31) should be approached cautiously when using the PAHQ in healthcare systems that differ from the Swiss system, and further validation is merited for other settings. 

Five of the six subscales had ‘acceptable’ to ‘excellent’ model reliability, while the ‘availability’ subscale had an alpha of 0.64, consistent with the original validation study by Hoseini-Esfidarjani et al. [21]. In the present study, all Cronbach’s alphas improved significantly compared with both the original study and the 6-factor model. Although Cronbach’s alpha is a widely used estimate for assessing scale reliability, it has been criticized in the past as an inadequate measure of reliability [54]. Cronbach’s alpha is affected by the number of items yet adding more items does not necessarily provide more meaningful information [55]. Moreover, multiple items measuring the same construct are highly correlated and assumed to assess the same latent variable, which may be inappropriate if unidimensionality is not ensured [56]. Accordingly, the present results on reliability should be interpreted with caution.

The significant correlations between the PAHQ subscales and the abilities outlined in the Levesque framework [14] underline the criterion-related validity of the PAHQ, particularly as these abilities have been identified as influential factors in access to care. These findings support the notion that access encompasses not only the individual characteristics of the person seeking care, such as demographic, economic, and social aspects, but also elements of their environment, including their healthcare provider and the broader healthcare system [10,14]. While previous research has typically focused on either access or the capabilities that facilitate access, rarely integrating these two perspectives, our study illustrates the value of bringing together patient and health system perspectives. This integrated approach is crucial for gaining a comprehensive understanding of access to care and suggests that future research should prioritize the collection of data from both perspectives [15].

### 4.1. Strengths and Limitations

This study provides insight into the psychometric properties of the PAHQ within the Swiss healthcare system and an RD patient population. Relative strengths of the study include the diverse, relatively large (for RDs) sample, and a strong theoretical foundation that offers applicability to a broader chronic disease context. In addition, we evaluated three models using CFA, providing a solid theoretical basis for the PAHQ. All the models have been widely utilized in the context of RDs lending support for using the PAHQ in the context of chronic and/or RDs. This theoretical basis contributes to a clearer definition of healthcare access.

This study has several limitations. Our data are exclusively based on the Swiss healthcare system, which may limit the generalizability of results. Future research should aim to replicate and validate the PAHQ in other health systems to assess its applicability in different settings. Second, the cross-sectional design neither enables assessment of test–retest reliability nor predictive validity over time. Future research could utilize longitudinal approaches to assess the measure’s stability and ability to predict healthcare access outcomes over time. While the two theoretical models used in the CFA analysis provide a robust theoretical foundation, it is unclear if these models can be further improved (or extended) for use in chronic disease populations. Future research could critically assess the applicability of these models to the specific needs of individuals with chronic diseases and consider potential adaptations to enhance their relevance. 

### 4.2. Implications 

The PAHQ can assess barriers and disparities in accessing healthcare and can be combined with data on interindividual ability measures [14]. Such validated instruments open promising avenues for intervention development addressing barriers to healthcare access. Findings can inform policy and initiatives promoting person-centered care that could ultimately lead to greater patient satisfaction and improved healthcare quality. The PAHQ can also be useful for identifying different disease-specific and sociodemographic factors that decrease healthcare access. Additionally, the PAHQ can be used to compare different populations (e.g., healthy vs. chronic disease) and healthcare systems. 

## 5. Conclusions

This study details the psychometric properties of the German version of the PAHQ in the context of individuals with RDs. The results demonstrate that the six-factor model, derived from the theoretical framework by Penchansky and Thomas [11], has a satisfactory fit (after removing six items). The findings suggest that the adapted PAHQ (Swiss RD population) has robust psychometric properties, including validity and reliability. These findings suggest that the PAHQ can effectively measure several dimensions of access to healthcare among people with RDs, thereby highlighting the challenges RD patients face in accessing care. The PAHQ can be a valuable tool for researchers and healthcare policymakers as it enables comparisons in access to care across different populations and health systems, including for people with chronic conditions such as RDs. Such functionality can inform resource allocation and intervention strategies to promote more equitable access to healthcare.

## Figures and Tables

**Table 1:** Sample characteristics (*N* = 271).

Sociodemographic & Disease-Related Variables	^1^ *N* (%)	^2^ *M* (*SD*)
Age (in years)		48.15 (15.32)
Gender		
Female	162 (59.78)	
Male	108 (39.85)	
Non-binary	1 (0.37)	
Education		
Less than nine years of education/special needs education	2 (0.74)	
Mandatory schooling time (nine years)	16 (5.90)	
Middle school/junior high school	99 (36.53)	
Senior high school/university of applied sciences	104 (38.378)	
University	50 (18.45)	
Marital Status		
Single	84 (31.00)	
Married	127 (46.86)	
Cohabiting or in a partnership	47 (17.34)	
Separated or divorced	15 (5.54)	
Widowed	4 (1.48)	
ICD-10 ^3^ disease type		
C00–D49, neoplasms	6 (2.21)	
D50–D89, diseases of the blood and blood-forming organs, and certain disorders involving the immune mechanism	23 (8.49)	
E00–E90, endocrine, nutritional, and metabolic diseases	58 (21.40)	
G00–G99, diseases of the nervous system	46 (16.97)	
H00–H59, diseases of the eye and adnexa	21 (7.75)	
I00–I99, diseases of the circulatory system	9 (3.21)	
K00–K93, diseases of the digestive system	28 (10.33)	
L00–L99, diseases of the skin and subcutaneous tissue	1 (0.37)	
M00–M99, diseases of the musculoskeletal system and connective tissue	11 (4.06)	
N00–N99, diseases of the genitourinary system	1 (0.37)	
Q00–Q99, congenital malformations, deformations, and chromosomal abnormalities	51 (18.82)	
S00–T98, injury, poisoning, and certain other consequences of external causes	9 (3.32)	
Disease type (according to ICD-11 ^3^)		
Developmental anomalies	7 (2.58)	
Disease course		
Stable	103 (38.01)	
Progressive	64 (23.62)	
Relapsing	67 (24.72)	
Improving	12 (4.43)	
Unknown	25 (9.23)	

^1^ *N* = sample size. ^2^ *M* = mean, and *SD* = standard deviation. ^3^ ICD = International Statistical Classification of Diseases and Related Health Problems.

**Table 2:** Chi-square test for the unidimensional-factor model, the five-factor model, and the six-factor model (*N* = 271).

Model	df ^1^	*χ* ^2 4^	*χ*^2^ Difference ^4^	*p*	AIC ^2^	BIC ^3^
Six-factor model	429	1087.014			19,841.15	20,194.16
Five-factor model	430	1556.793	92.70	<0.001	20,308.93	20,658.34
Unidimensional-factor model	435	4184.625	753.98	<0.001	22,926.76	23,258.16

^1^ df = degrees of freedom. ^2^ AIC = Akaike information criterion. ^3^ BIC = Bayesian information criterion. ^4^ *χ*^2^ = Chi-square.

**Table 3:** Cronbach’s alpha (α) of subscales for the reduced and original six-factor model [11].

	Reduced Six-Factor Model	Original Six-Factor Model
Latent Factor	α	α
Accessibility	0.91	0.83
Item 1		0.91
Item 2	0.83	0.74
Item 3	0.80	0.72
Item 4	0.96	0.75
Availability	0.64	0.64
Item 5	0.60	0.60
Item 6	0.35	0.35
Item 7	0.64	0.64
Acceptability	0.89	0.86
Item 8	0.88	0.84
Item 9	0.87	0.83
Item 10	0.86	0.82
Item 11	0.87	0.83
Item 12	0.88	0.84
Item 13		0.87
Item 14		0.88
Item 15	0.88	0.84
Item 16	0.89	0.85
Affordability	0.75	0.45
Item 17	0.70	−0.041
Item 18	0.52	0.101
Item 19		0.749
Adequacy	0.77	0.76
Item 20	0.69	0.70
Item 21	0.69	0.70
Item 22	0.77	0.76
Item 23	0.74	0.73
Item 24	0.71	0.71
Item 25		0.77
Awareness	0.84	0.82
Item 26	0.79	0.77
Item 27	0.82	0.79
Item 28	0.80	0.78
Item 29	0.77	0.76
Item 30	0.84	0.80
Item 31		0.84

**Table 4:** Ceiling and floor effects of subscales for the reduced six-factor model [11].

Latent Factor	Floor Effect, %	Ceiling Effect, %
Accessibility	1.11	21.77
Availability	0.37	8.12
Acceptability	1.48	6.64
Affordability	0.74	25.46
Adequacy	0.37	3.32
Awareness	0.37	5.90

**Table 5:** Pearson correlations of the Ability Scales and the revised PAHQ.

Variable	*M* ^1^	*SD* ^2^	1	2	3	4	5	6	7	8	9	10
1. Accessibility	11.45	2.92										
2. Availability	11.24	2.22	0.43 **									
			[0.32, 0.52]									
3. Acceptability	27.04	4.92	0.32 **	0.66 **								
			[0.21, 0.42]	[0.59, 0.72]								
4. Affordability	7.93	1.88	0.12 *	0.17 **	0.14 *							
			[0.00, 0.24]	[0.05, 0.28]	[0.02, 0.25]							
5. Adequacy	17.86	3.46	0.38 **	0.59 **	0.64 **	0.22 **						
			[0.27, 0.47]	[0.50, 0.66]	[0.56, 0.71]	[0.11, 0.33]						
6. Awareness	18.92	3.59	0.25 **	0.45 **	0.68 **	0.07	0.61 **					
			[0.14, 0.36]	[0.35, 0.54]	[0.61, 0.74]	[−0.05, 0.19]	[0.53, 0.68]					
7. API ^3^	11.14	4.01	0.11	0.25 **	0.25 **	0.24 **	0.19 **	0.13 *				
			[−0.00, 0.23]	[0.14, 0.36]	[0.13, 0.35]	[0.13, 0.35]	[0.08, 0.31]	[0.02, 0.25]				
8. HLS-Q12 ^4^	33.90	4.87	0.13 *	0.25 **	0.26 **	0.07	0.25 **	0.38 **	0.01			
			[0.01, 0.24]	[0.13, 0.36]	[0.14, 0.36]	[−0.05, 0.19]	[0.13, 0.35]	[0.28, 0.48]	[−0.11, 0.13]			
9. PAM13 ^5^	41.12	5.39	0.17 **	0.21 **	0.22 **	0.18 **	0.27 **	0.26 **	0.05	0.46 **		
			[0.06, 0.29]	[0.10, 0.32]	[0.10, 0.33]	[0.06, 0.29]	[0.16, 0.38]	[0.15, 0.37]	[−0.07, 0.17]	[0.36, 0.55]		
10. SF-12 PCS ^6^	359.50	176.15	0.36 **	0.32 **	0.23 **	0.05	0.19 **	0.18 **	0.14 *	0.22 **	0.22 **	
			[0.25, 0.46]	[0.20, 0.42]	[0.11, 0.34]	[−0.07, 0.16]	[0.08, 0.30]	[0.07, 0.30]	[0.02, 0.25]	[0.10, 0.33]	[0.10, 0.33]	
11. Education	4.54	1.10	0.05	−0.03	−0.07	−0.12 *	−0.07	0.08	−0.20 **	0.13 *	0.00	0.11
			[−0.07, 0.17]	[−0.14, 0.09]	[−0.19, 0.05]	[−0.24, −0.00]	[−0.19, 0.05]	[-0.04, 0.20]	[−0.31, −0.08]	[0.02, 0.25]	[−0.12, 0.12]	[−0.01, 0.23]

^1^*M* = mean. ^2^ *SD* = Standard deviation. ^3^ API = Autonomy Preference Index. ^4^ HLS-Q12 = 12-item European Health Literacy Survey. ^5^ PAM13 = Patient Activation Measure. ^6^ SF-12 PCS = Short Form Health Survey 12 Items Physical Component Summary. * *p* < 0.05; ** *p* < 0.01.

## Data Availability

The datasets used and/or analyzed during the current study are available from the corresponding author upon reasonable request.

## Supplementary Materials

The following supporting information can be downloaded at: https://www.mdpi.com/article/10.3390/healthcare12060661/s1, Table S1: PAHQ questionnaire items; Table S2: Power analysis for structural equation models (SEM); Table S3: Factor loadings of six-factor solution after confirmatory factor analysis; Table S4: Factor loadings of six-factor solution following item removal.
