# Supplementary material for: Psychometric Evaluation of the German Version of the Perceived Access to Healthcare Questionnaire in a Sample of Individuals with Rare Chronic Diseases
Source: Healthcare (Basel). 2024 Mar 15;12(6):661. doi: 10.3390/healthcare12060661 (PMC10970657; doi:10.3390/healthcare12060661)
Supplement: Supplementary file 1 [file healthcare-12-00661-s001.zip › healthcare-2887661-supplementary.pdf]

Table S1

PAHQ questionnaire items

Latent variables	Indicator	Items in English	Items in German
Accessibility	1	The medical care I need is provided at healthcare facilities.	Die medizinische Versorgung, die ich benötige, wird in Gesundheitseinrichtungen angeboten.
	2	The distance between healthcare facilities and my home is appropriate.	Die Entfernung zwischen Gesundheitseinrichtungen und meinem Wohnort ist angemessen.
	3	The time it takes me to reach healthcare facilities is appropriate.	Die Zeit, welche ich benötige, um Gesundheitseinrichtungen zu erreichen, ist angemessen.
	4	Getting to and from healthcare facilities is easy for me.	Der Hin- und Rückweg von den Gesundheitseinrichtungen ist für mich einfach zu bewältigen.
Availability	5	The medical care I need (vaccinations, doctor visits, family planning, maternal and child care, injections, etc.) is provided at public health facilities.	Die von mir benötigte medizinische Versorgung (Impfungen, Arztbesuche, Familienplanung, Betreuung von Mutter und Kind, Injektionen usw.) wird in öffentlichen Gesundheitseinrichtungen angeboten.
	6	The equipment of the healthcare facilities meets the health needs of the	Die Ausstattung der Gesundheitseinrichtungen entsprechen den

		patients.	gesundheitlichen Bedürfnissen der Patient:innen.
	7	The healthcare staff is adjusted to the number of patients and their needs.	Das Gesundheitspersonal ist auf die Anzahl der Patient:innen und deren Bedürfnisse abgestimmt.
Acceptability	8	The quality of medical care offered at healthcare facilities is acceptable.	Die Qualität der im Gesundheitseinrichtungen angebotenen medizinischen Versorgung ist akzeptabel.
	9	Healthcare staff addresses the needs of patients, for example, by familiarizing them with available resources in their surroundings.	Das Personal der Gesundheitseinrichtungen geht auf die Bedürfnisse der Patient:innen ein, indem es sie z. B. mit den verfügbaren Ressourcen der Umgebung vertraut macht.
	10	The healthcare staff listens carefully to what I have to say.	Die Mitarbeiter des Gesundheitspersonals hören aufmerksam zu, was ich zu sagen habe.
	11	The healthcare staff gives me enough time.	Das Gesundheitspersonal lässt mir genügend Zeit.
	12	I trust the statements of the treatment team (doctor, nurse, midwife, etc.) regarding my health and illness.	Ich vertraue den Aussagen des behandelnden Teams (Arzt, Pflegepersonal, Hebamme, etc.) bezüglich meiner Gesundheit und Krankheit.

	13	My request for same-sex healthcare staff is being considered.	Meinem Wunsch nach gleichgeschlechtlichem Gesundheitspersonal wird Beachtung geschenkt.
	14	I accept screenings such as cervical and colorectal cancer at healthcare facilities.	Ich akzeptiere Vorsorgeuntersuchungen wie Gebärmutterhals- und Darmkrebs in Gesundheitseinrichtungen.
	15	The treatment team at the healthcare facilities is respectful.	Das behandelnde Team in den Gesundheitseinrichtungen ist respektvoll.
	16	Healthcare staff (doctors, nurses, midwives, etc.) are familiar with the culture of patients and communicate with them appropriately.	Das Gesundheitspersonal (Ärzte, Pflegepersonal, Hebammen etc.) ist mit der Kultur der Patient:innen vertraut und kommuniziert dementsprechend mit ihnen.
Affordability	17	To solve a health problem, I first go to a family doctor/general practitioner.	Um ein gesundheitliches Problem zu lösen, gehe ich zuerst zu einem Hausarzt/Allgemeinmediziner.
	18	I am referred to specialized professionals by my general practitioner/family physician.	Von meinem Allgemeinmediziner/Hausarzt werde ich zu spezialisierten Fachkräften weitergeleitet.
	19	Cost is a major barrier to healthcare utilization.	Kosten sind ein wesentliches Hindernis für die Inanspruchnahme der Gesundheitsversorgung.

Accommodation	20	It is easy to make an appointment at healthcare facilities.	Es ist einfach, einen Termin in Gesundheitseinrichtungen zu vereinbaren.
	21	The expected time to receive the medical care I need is appropriate.	Die zu erwartende Zeit bis zum Erhalt der benötigten medizinischen Versorgung ist angemessen.
	22	I can discuss health issues and changes in my condition directly with the team treating me (doctor, nurse, midwife, etc.) by phone.	Ich kann Gesundheitsfragen und Änderungen meines Zustands telefonisch direkt mit dem behandelnden Team (Arzt, Pflegepersonal, Hebamme etc.) besprechen.
	23	The working hours of public health facilities are suitable for seeking medical care.	Die Arbeitszeiten der öffentlichen Gesundheitseinrichtungen sind für die Inanspruchnahme von medizinischer Versorgung geeignet.
	24	The physical space of the healthcare facilities is suitable for the utilization of medical care.	Die Räumlichkeiten der Gesundheitseinrichtungen sind für die Inanspruchnahme von medizinischer Versorgung geeignet.
	25	Healthcare facilities provide access to various assistive devices such as wheelchairs, walkers and the like.	Die Gesundheitseinrichtungen bieten Zugang zu verschiedenen Hilfsmitteln wie Rollstühlen, Gehhilfen und dergleichen.

Awareness	26	The information I receive is prepared in such a way that I understand it.	Die Informationen, die ich erhalte, sind so aufbereitet, dass ich sie verstehe.
	27	The information I need is expressed in understandable language and without the use of specialized terminology.	Die Informationen, die ich benötige, werden in verständlicher Sprache und ohne die Verwendung von Fachbegriffen formuliert.
	28	Communication of healthcare staff (physician, nurse, midwife, etc.) with patients is appropriate.	Die Kommunikation des Gesundheitspersonals (Arzt, Pflegepersonal, Hebamme usw.) mit den Patienten ist angemessen.
	29	The healthcare staff tries to make sure I fully understand the health information provided.	Das Gesundheitspersonals versucht sicherzustellen, dass ich die zur Verfügung gestellten Gesundheitsinformationen vollständig verstehe.
	30	My life circumstances are taken into account, such as marital status, financial possibilities and cultural differences.	Meine Lebensumstände werden berücksichtigt, wie Familienstand, finanzielle Möglichkeiten und kulturelle Unterschiede.
	31	My life circumstances are taken into account, such as marital status, financial possibilities and cultural differences.	Ich habe eine medizinische Fachkraft, bei der alle meine Gesundheitsinformationen zusammenkommen.

Table S2

Power analysis for structural equation models (SEM)

**Model**	**Number of latent**	**Number of observed variables**	Minimum sample size to detect effects	Number of observed variables	Recommended minimum sample size
Prior to item exclusion					
Six-factor model	6	31	161	110	161
Five-factor model	5	31	150	233	233
Unidimensional -factor model	1	31	10	35.2	35.2
Following item exclusion					
Six-factor model	6	25	161	94	161

Notes. Calculations were based on the calculator by Daniel Soper: https://www.danielsoper.com/statcalc/calculator.aspx?id=89

Table S3

Factor loadings of six-factor solution after confirmatory factor analysis

							95% CI	
Latent factor	Item number	*B*	*SE*	*z*	*p*	β	*LL*	*UL*
Accessibility	1	0.335	0.065	5.125	0.000	0.325	0.207	0.335
	2	0.914	0.048	19.044	0.000	0.938	0.820	0.914
	3	0.938	0.046	20.458	0.000	0.980	0.848	0.938
	4	0.756	0.050	14.979	0.000	0.730	0.657	0.756
Availability	5	0.207	0.064	3.254	0.001	0.501	0.082	0.207
	6	0.258	0.077	3.359	0.001	0.675	0.107	0.258
	7	0.306	0.067	4.564	0.000	0.666	0.175	0.306
Acceptability	8	0.204	0.034	5.970	0.000	0.697	0.137	0.204
	9	0.280	0.050	5.564	0.000	0.770	0.181	0.280
	10	0.301	0.059	5.136	0.000	0.835	0.186	0.301
	11	0.297	0.056	5.326	0.000	0.799	0.188	0.297
	12	0.282	0.050	5.696	0.000	0.756	0.185	0.282
	13	0.071	0.022	3.206	0.001	0.276	0.028	0.071
	14	0.087	0.029	3.001	0.003	0.253	0.030	0.087
	15	0.204	0.038	5.368	0.000	0.722	0.130	0.204
	16	0.185	0.037	5.021	0.000	0.607	0.113	0.185

Latent factor	Item number	*B*	*SE*	*z*	*p*	β	95% CI *LL*	95% CI *UL*
Affordability	17	0.658	0.070	9.450	0.000	0.605	0.521	0.658
	18	0.939	0.045	20.900	0.000	1.000	0.851	0.939
	19	0.026	0.080	0.325	0.745	0.021	-0.131	0.026
Accommodation	20	0.393	0.075	5.263	0.000	0.705	0.247	0.393
	21	0.386	0.070	5.553	0.000	0.718	0.250	0.386
	22	0.244	0.044	5.573	0.000	0.476	0.158	0.244
	23	0.253	0.043	5.912	0.000	0.571	0.169	0.253
	24	0.261	0.037	7.125	0.000	0.702	0.190	0.261
	25	0.172	0.035	4.858	0.000	0.408	0.103	0.172
Awareness	26	0.360	0.076	4.719	0.000	0.700	0.210	0.360
	27	0.338	0.076	4.446	0.000	0.616	0.189	0.338
	28	0.370	0.044	8.435	0.000	0.802	0.284	0.370
	29	0.414	0.065	6.410	0.000	0.805	0.288	0.414
	30	0.361	0.047	7.747	0.000	0.637	0.270	0.361
	31	0.265	0.051	5.243	0.000	0.392	0.166	0.265
Accessibility	Factor 1	0.378	0.076	4.946	0.000	0.353	0.228	0.378
Availability	Factor 2	2.089	0.583	3.582	0.000	0.902	0.946	2.089
Acceptability	Factor 3	2.465	0.519	4.752	0.000	0.927	1.448	2.465
Affordability	Factor 4	0.250	0.077	3.241	0.001	0.243	0.099	0.250

Latent factor	Item number	*B*	*SE*	*z*	*p*	β	95% CI *LL*	95% CI *UL*
Accommodation	Factor 5	1.699	0.308	5.506	0.000	0.862	1.094	1.699
Awareness	Factor 6	1.463	0.285	5.127	0.000	0.826	0.904	1.463

Notes. *B* = unstandardized loadings. *SE* = standard error. β = standardized loadings. *z* = z-value. *p* = p-value. CI = confidence interval. LL = lower limit. UL = upper limit. HCA = healthcare access.

Table S4

Factor loadings of six-factor solution following item removal

Latent factor	Item number	*B*	*SE*	*z*	*p*	β	95% CI *LL*	95% CI *UL*
Accessibility	2	0.910	0.048	19.081	0.000	0.932	0.816	0.910
	3	0.946	0.047	20.294	0.000	0.988	0.855	0.946
	4	0.752	0.051	14.856	0.000	0.724	0.653	0.752
Availability	5	0.201	0.064	3.124	0.002	0.497	0.075	0.201
	6	0.252	0.078	3.244	0.001	0.675	0.100	0.252
	7	0.301	0.069	4.368	0.000	0.669	0.166	0.301
Acceptability	8	0.209	0.034	6.144	0.000	0.699	0.142	0.209
	9	0.287	0.050	5.698	0.000	0.772	0.188	0.287
	10	0.310	0.059	5.249	0.000	0.840	0.194	0.310
	11	0.305	0.056	5.440	0.000	0.802	0.195	0.305
	12	0.287	0.049	5.821	0.000	0.754	0.191	0.287
	15	0.207	0.037	5.539	0.000	0.716	0.134	0.207
	16	0.187	0.036	5.151	0.000	0.600	0.116	0.187
Affordability	17	1.000	0.000	NA	NA	0.738	1.000	1.000
	18	1.000	0.000	NA	NA	1.000	1.000	1.000

							95% CI	
Latent factor	Item number	*B*	*SE*	*z*	*p*	β	*LL*	*UL*
Accommodation	20	0.424	0.084	5.045	0.000	0.723	0.260	0.424
	21	0.417	0.078	5.330	0.000	0.738	0.264	0.417
	22	0.255	0.045	5.693	0.000	0.472	0.167	0.255
	23	0.262	0.043	6.031	0.000	0.560	0.177	0.262
	24	0.271	0.036	7.422	0.000	0.690	0.199	0.271
Awareness	26	0.370	0.079	4.664	0.000	0.705	0.215	0.370
	27	0.344	0.079	4.357	0.000	0.613	0.189	0.344
	28	0.381	0.044	8.703	0.000	0.807	0.295	0.381
	29	0.425	0.066	6.396	0.000	0.807	0.295	0.425
	30	0.367	0.047	7.808	0.000	0.632	0.275	0.367
Accessibility	Factor 1	0.373	0.076	4.888	0.000	0.349	0.223	0.373
Availability	Factor 2	2.144	0.622	3.447	0.001	0.906	0.925	2.144
Acceptability	Factor 3	2.407	0.499	4.827	0.000	0.923	1.430	2.407
Affordability	Factor 4	0.228	0.071	3.210	0.001	0.222	0.089	0.228
Accommodation	Factor 5	1.583	0.305	5.187	0.000	0.845	0.985	1.583
Awareness	Factor 6	1.414	0.282	5.025	0.000	0.817	0.863	1.414

Notes. *B* = unstandardized loadings. *SE* = standard error. β = standardized loadings. *z* = z-value. *p* = p-value. CI = confidence interval. LL = lower limit. UL = upper limit. NA = not available. HCA = healthcare access.
